# Biochemical Profile and Antioxidant Properties of Propolis from Northern Spain

**DOI:** 10.3390/foods12234337

**Published:** 2023-12-01

**Authors:** Eugenia Rendueles, Elba Mauriz, Javier Sanz-Gómez, Ana M. González-Paramás, María-E. Vallejo-Pascual, Félix Adanero-Jorge, Camino García-Fernández

**Affiliations:** 1 Institute of Food Science and Technology (ICTAL), La Serna 58, 24007 León, Spain; jjsang@unileon.es (J.S.-G.); mc.garcia@unileon.es (C.G.-F.); 2 ALINS, Food Nutrition and Safety Investigation Group, Universidad de León, 24007 León, Spain; 3 GIP-USAL, Polyphenol Investigation Group, Universidad de Salamanca, 37007 Salamanca, Spain; paramas@usal.es; 4 Quantitative Methods Area, Economical and Statistical Department, Universidad de León, 24007 León, Spain; mevalp@unileon.es

**Keywords:** propolis, honeybee products, bioactive compounds, antioxidant properties, food industry

## Abstract

The antioxidant, anti-inflammatory, and antimicrobial characteristics of propolis, a bioactive compound collected from hives, have prompted its use in the food sector in recent times. This study investigated the physicochemical characteristics, phenolic profile, and antioxidant capacity of 31 propolis extracts collected from Northern Spain. The physicochemical composition (resins, waxes, ashes mineral content, and heavy metals) was within the allowable regulatory limits. The analysis of bioactive compounds enabled the identification of 51 constituents: flavonoids (apigenin, catechin, chrysin, quercetin, and pinocembrin) and phenolic acids (caffeic, ferulic, and coumaric). The mean value of total polyphenols was 42.72 ± 13.19 Pinocembrin–Galangin Equivalents/100 g, whereas a range between 1.64 ± 0.04 and 4.95 ± 0.36 Quercetin Equivalents (QE) g/100 g was found for total flavonoids content. The determination of bioactivities revealed significant antioxidant capacity using DPPH (1114.28 ± 10.39 µM Trolox Equivalents and 3487.61 ± 318.66 µM Vitamin C Equivalents). Resin content in propolis samples was positively and significantly correlated with both polyphenols (rho = 0.365; *p* = 0.043) and flavonoid composition (rho = 0.615; *p* = 0.000) as well as the antioxidant capacity TEAC DPPH (rho = 0.415; *p* = 0.020). A multiple regression analysis modeled the correlation between resin composition, flavonoids, and TEAC DPPH values, yielding a significant regression equation (R^2^ = 0.618; F (2,28) = 22.629; *p* < 0.000; d = 2.299). Therefore, evaluating physicochemical parameters and biological activities provides a promising framework for predicting propolis’ quality and antioxidant properties, thus suggesting its potential as a functional and bioactive compound for the food industry.

## 1. Introduction

Most researchers describe propolis as a resinous or sticky substance that bees collect from various plants, mix with waxes, and use as a construction material in their hive [1,2,3]. In the present, across the planet, numerous references, investigations, citations, and innovations in the use of this honey-derived product, and thus the antimicrobial, antioxidant, anti-inflammatory, lately, antitumor, immunomodulatory, and biological marker properties, have been unearthed and certified [4,5,6,7]. At the same time, the complexity of its chemical composition (thanks to chromatographic techniques) and its botanical origin have become better known.

A possible mechanism of social immunity of colony strength has been verified that propolis directly affects the microbial load of the hive, influencing the reduction in the expression of the bee’s immunity, regardless of the level of pathogens or parasites in the hive [8,9]. It has been observed that the European honeybee (*A. mellifera*) uses these “resins” to clean, insulate, and reinforce the hive, covering holes and cracks, reducing bites, thus maintaining homeostatic balance, inhibiting microbial growth, controlling airflow, waterproofing the walls against humidity, and creating some protection against external invaders, such as the greater wax moth [10] (*Galleria mellonella* Linnaeus, 1758) or the small hive beetle (*Aethinia tumida* Murray, 1867). Simone-Finstrom and Spivak [11] believe that there is a genetic component in bees that determines that some of them collect “resins” and also stimuli such as holes, cracks, irregularities within the hive, or the time it takes for cementing bees to unload “resins” from foragers to make propolis.

Studies carried out by various authors have made it possible to identify, through different chromatographic techniques, the same components present in plants and in propolis, thus establishing a relationship in terms of their botanical and geographical origin [12,13]. In this regard, it is essential to indicate that plant variability directly influences the chemical composition, resulting in an inhomogeneous product and, therefore, making it difficult to standardize [14,15]. Thus, when consulting the bibliography, descriptions of types of propolis are found that are identified by their chemical profile, such as “Poplar type”, “Birch type”, “Tropical type”, “Mediterranean type”, and “Pacific type” [1,16,17,18].

It has been maintained that the fundamental constituents always present in the composition of propolis are resins (oleo-gum resins), beeswax, insoluble impurities, pollen, and spores [12,19]. The polyphenolic compounds primarily found in the resinous fraction stand out as characteristic and typical of each type of propolis. Propolis has become a priceless natural product that provides access to plant metabolites, which would be hard to detect in the plant biodiversity without damaging them [20,21].

Another aspect to consider in the analysis of propolis is the mineral content, which contributes to improving its nutritional value. While minerals are related to botanical origin, the quantity of heavy metals depends significantly on hive management [13,22]. In this sense, the World Health Organization (WHO) included Fe, Cd, Pb, and Hg in the list of the 10 chemical products that cause serious public health problems and set a maximum level in food through the Codex Alimentarius [23]. The pollens found in the propolis result from pollen from the flora around the hives, together with the “contamination” of aerial pollens that arrive and settle, impregnating the resin and transferring it to the product [24,25].

Regarding its biological properties, these depend on the type of flavonoid. Numerous studies on propolis show the beneficial properties of phenolic compounds, especially flavonoids [26,27]. Phenolic compounds, with more than 8000 reported structures, constitute a vast group in the plant kingdom. This group includes simple phenols, flavonoids, and low-molecular-weight tannins, such as gallic acid, caffeic acid, p-coumaric acid, ellagic acid, transferulic acid, vanillin, caffeic acid esters (CAPE and isoprenyl caffeate), quercetin, and apigenin, among others [8,28,29,30]. Flavonoids represent nature’s most essential and abundant group of phenolic compounds, with more than 6000 identified structures.

In Spain, propolis is authorized as a food supplement; it is found on the market in multiple presentations, made with different types of propolis in which the main active compounds are not identified and with information on the label without any criteria regarding the doses indicated [9,31]. At the European level, the EFSA issued a scientific opinion in 2010 [32] stating that propolis’ possible beneficial effects on health were directly related to their composition and origin, along the lines previously shown by other authors [3,33,34].

In addition to its antioxidant effect, the use of propolis in the food industry is justified by various authors as a preservative [35,36], thanks to its antibacterial and antifungal action that some extracts have on pathogenic and altering microorganisms of interest in this industry [37,38,39]

The aim of this study is to evaluate the total characterization of propolis collected around two harvests in the north of Spain. Knowing these products’ different capacities and properties when used in the food industry could be interesting. There is a possibility of finding an economical option for beekeepers to implement the rentability of the harvesting sector while the propolis improves its economic value.

## 2. Materials and Methods

### 2.1. Materials and Reagents

All the extraction assays used ethanol from Panreac and methanol (Labkem, Barcelona, Spain). The equipment used for the different determinations and analyses were Soxtec System HT 1043 (Tecator, DK-3400 Hilleroed, Denmark) and Muffle Oven Mod. 10PR/300 Serial 88 (Hobersal, Barcelona, Spain), ICP-MS NexION 300D (Perkin-Helmer, Waltham, MA, USA), Centrifuge 5810 R (Eppendorf, Hamburg, Germany), Hewlett-Packard 1200 (Agilent Technologies, Waldbronn, Germany), Mass Spectrophotometer (Applied Biosistems 3200 Q TRAP LC/MS/MS System, Waltham, MA, USA), SpectraMax iD3 (Molecular Devices, San José, CA, USA), and Spectrophotometer DU 7400 (Beckman, Brea, CA, USA). The preparation of the calibration graph involved the following products from different trading houses: Pinocembrin, Med Chem Express, and Galangin, Med Chem Express, both from Sweden and Quercetin, Sigma Aldrich (St. Louis, MO, USA). Different homologated and commercial kits were used: Antioxidant Activity Kit (ABTS Antioxidant Capacity Batch 10022604 and DPPH Antioxidant Capacity Batch 10072004. BQC Redox Technologies. Asturias, Spain). Optical Microscope (OM) Nikon Eclipse 80i and Scanning Electronic Microscope JSM 6480 mod JEOL were used.

### 2.2. Groups of Propolis and Sample Collection

This study continued a previous investigation that evaluated the effect of antibacterial activity against *Listeria* [40]. A total of 31 samples of propolis were collected from several geographical areas in Spain throughout 2019 and 2020. Beekeepers from different regions of Spain voluntarily participated in this study, collecting propolis samples according to their usual management practices. Macroscopically, differences were observed among raw samples: texture, color, and compactness degree. Propolis was stored in freezing conditions (−20 °C) from collection to analysis.

### 2.3. Preparation of Propolis Extracts

Propolis extraction of pulverized raw propolis (10 g) comprised the addition of 600 mL of 70% hydroalcoholic solution. The extraction was carried out in two stages of 24 h each of shaking at a controlled temperature of 20 °C of the 10 g of propolis sample, first in 300 mL of 70% ethanol and after filtering a new extraction under the same conditions of shaking time and temperature [20]. Ethanol extracts of propolis (EEP) were kept at a refrigerated temperature (4 °C) from that moment and throughout the procedure to keep their properties intact.

### 2.4. Characterization of Propolis Samples

EEPs were analyzed to determine the main components responsible for their quality, as well as their bioactivity and chemical properties. The characterization of the selected propolis samples considered the proximal composition regardless of their botanical origin, including resin, water, wax, impurity, and ash content [2,14,41].

#### 2.4.1. Wax Determination

According to procedures described by Bankova et al. [14,42], 1 g of the powdered raw propolis (triplicate) was weighed then treated with n-hexane in Soxhlet for 2 h using a weighted cartridge. Then, cartridges were introduced in an oven at 105 °C for 10 min to eliminate the n-hexane and cool them until weighed again.

#### 2.4.2. Mechanical Impurities Content

The rest of the cartridge content (propolis sample) after the procedure described in Section 2.4.1. was resuspended in 100 mL of ethanol at 70%. This procedure was conducted by shaking for 24 h at 20 °C. Then, the weighted cartridge was transferred together with the residue and, using a weighted filter disc and a vacuum bomb, the total liquid was retired in a flask. The rest of the impurities were dried in a desiccator until constant weight [14].

#### 2.4.3. Resin Content

Before the wax extraction, from the extract diluted in 100 mL of ethanol 70%, 20 mL were weighed and then concentrated in a rotary evaporator to obtain a solid residue; after cooling, the concentrates were weighed again, expressing the results as % *w*/*w* [14].

#### 2.4.4. Ash Content Determination

The ash content was determined using the AOAC method [43]. A homogeneous sample of 1 g of pulverized crude propolis (PBP) was taken to a tared porcelain capsule and 0.1 mL of hydrogen peroxide 33% and placed for precalcination for 2–3 h. Afterward, the sample was completely calcined until white ashes were obtained according to the method recommendations. At the end, the % ASH was calculated as follows: weight (capsule + ashes) (g) − weight (empty capsule) (g) = weight of ashes (g), % ASH PBP = weight of ashes (g) × 100/initial weight.

#### 2.4.5. Total Mineral and High Metal Determination

For the analysis of the elements, the propolis samples were digested (0.500 g of each sample in 10 mL of 65% nitric acid and 3 mL of 37% hydrochloric acid) in a digester at atmospheric pressure with reflux programmed with the following temperature and time conditions: 20 to 45 °C in 30 min, 1 min at 45 °C, from 45 to 65 °C in 25 min, 5 min at 65 °C, from 65 to 100 °C in 15 min, and 120 min at 100 °C. The digestion tubes were allowed to cool, and the digestion tubes were volumetrically filled to 50 mL with MilliQ water. Pb, Cd, and Hg were measured on an ICP-MS. For this purpose, the digestions were diluted 1:4 in MilliQ water, and 10 ppb of Pt and Rh were added as internal standards. The ICPMS equipment was calibrated with Pb and Cd standards of 2, 10, 50, and 100 ppb and with Hg standards of 0, 2, 10, and 20 ppb in nitric acid diluted 1:20 (*v*/*v*) to which 10 ppb of Pt and Rh were also added as internal standards. The rest of the elements were analyzed in an ICPOES using the dilutions calibrated to 50 mL without dilution. The equipment was calibrated with standards of 0,1, 1, 10, and 20 ppm in nitric acid 1:5 (*v*/*v*), except for Na, which was used 1, 10, and 20 ppm. As an internal standard, 5 ppm of Y was used.

#### 2.4.6. Palynological Composition

From the homogeneous sample kept at freezing, 0.5 g was taken and placed in an Erlenmeyer flask together with 15 mL of 96° ethanol and left for 24 h at room temperature. The sample was submitted to several steps of centrifugation, decantation, and digestion with KOH 10%. Finally, the supernatant was removed and the residue was deposited in Eppendorf tubes to which 3–4 drops of phenol water were added, and the samples were kept refrigerated for subsequent mounting. The samples were observed by optical microscopy and SEM. A minimum of 300 pollen grains per sample were identified [44] using as reference the palynological library of the Botany Department of the University of León, as well as various atlases and pollen identification keys and the online pollen resource of the Society for the Promotion of Palynological Research in Austria (2017).

### 2.5. Determination of Total Polyphenol Content

Total polyphenol content (PTC) was analyzed according to Folin–Ciocalteu’s method (as modified by Bankova et al. [14]). The calibration graph was prepared with standard methanolic solutions of a mixture of pinocembrin–galanin at 2:1 (*w*/*w*) in a 25–300 μg/mL range. A total of 0.5 mL of the 31 propolis extracts was transferred in triplicate into a 25 mL volumetric flask. Then, 7.5 mL of distilled water, 2 mL of the Folin–Ciocalteu’s regent, and 3 mL of a 20% N_2_CO_3_ solution were added. The volume was filled to 25 mL with distilled water and left at room temperature for 2 h. Absorbance measurements were performed in triplicate at 760 nm in a UV-vis spectrophotometer, expressing the results obtained as grams of pinocembrin–galangin equivalents per 100 g of raw propolis (%PGE) [14,45].

### 2.6. Determination of Total Flavonoids, Flavone, and Flavonol Content

Total flavonoid content (TFC) was estimated according to Woisky and Salatino [46], using quercetin as standard and expressing the results obtained as grams of quercetin equivalents per 100 g of raw propolis (%QE). Absorbance measurements at 415 nm after 40 min of incubation in the dark at room temperature against a blank were performed on a spectrophotometer [14,41]. Flavone and flavonol content were determined using the spectrophotometric method proposed by Bankova et al. [14] based on the reaction to form aluminum chloride complexes. The calibration line preparation involved a methanolic solution of galangin. From the ethanolic extract of propolis, 0.5 mL was used and 4.3 mL of 70% ethanol, 0.21 mL of Al(NO_3_)_3_, and 0.10 mL of 1 M CH_3_CO_2_K were added. This was mixed well and at least 40 min/24 °C. Absorbance measurements were performed in triplicate at 425 nm in a UV-vis spectrophotometer. The results obtained are expressed as grams of galangin equivalents per 100 g of raw propolis (%GE).

### 2.7. HPLC Determination

Previously, for HPLC analysis, 100 mg of raw propolis was pretreated to remove different impurities and substances that may interfere with the determination. Then, a similar process was used to extract phenolic compounds. In this case, the extraction was performed in triplicate, and the result was dried and dissolved in 60% methanol [47]. Once the extract was obtained and before its analysis, it was necessary to perform a filtration process (ClarinetTM, Hydrophilic PVDF 0.45 µm, Agela Technologies, Torrance, CA, USA) to be injected into the chromatograph. The analysis of phenolic compounds was performed by reversed-phase high-performance liquid chromatography, using online double detection by diode array spectrophotometer–mass spectrometry (HPLC-DAD-MS). The chromatographic equipment was a Hewlett-Packard 1200 (Agilent Technologies, Waldbronn, Germany) equipped with a binary pump and a diode array detector coupled to the HP Chem Station (rev. A.05.04). The separation was conducted on a Phenomenex Aqua^®^ C18 column (5 μm, 150 mm × 4.6 mm) thermostatic at 35 °C, using 0.1% formic acid (eluent A) and acetonitrile (eluent B) as mobile phase. A flow rate of 0.5 mL/min was set, establishing the elution gradient. The injection volume was 15 μL and spectrophotometric detection was performed by selecting 280, 330, and 360 nm as preferred wavelengths. Mass analysis was conducted using the mass spectrometer, operating in negative ionization mode at a temperature of 400 °C and recording spectra between *m*/*z* 100 and *m*/*z* 1000. Zero air was used as nebulizer gas (30 psi) and turbo gas (400 °C, 40 psi) for eluent drying and nitrogen as curtain gas (20 psi) and medium collision gas [48].

The detection method employed was full scan at high sensitivity (Enhanced MS, EMS) with the following parameters: capillary voltage, −4500 V with the following potentials: declustering potential (DP) −50 V, entrance potential (EP) −6 V, and collision energy (CE) −10 V. Following this analysis, another analysis was carried out in Enhanced Product Ion (EPI) mode to obtain the characteristic fragmentation of the majority ion obtained in the first experiment. In this case, the conditions used were DP −50 V, EP −6 V, CE −25 V, and collision energy spread (CES) 0 V. The phenolic compounds were identified based on the retention time criteria observed in the chromatograms, the UV-visible spectra, and the MS and MSn data obtained in the mass spectrometer, comparing the different data with those available in the literature.

### 2.8. Antioxidant Properties

BQC DPPH TAC Assay Kit is based on the 2,2-diphenyl-1-picrylhydrazyl (DPPH) method. In this method, the DPPH free radical (DPPH•), a deep purple-colored (λ_max_ = 517 nm) stable organic nitrogen radical, is reduced by antioxidants to the colorless DPPH reduced form. Therefore, the absorbance decrease at 517 nm depends linearly on the antioxidant concentration. The synthetic antioxidant Trolox is used to standardize the sample TAC relative to Trolox (Trolox Equivalent Antioxidant Capacity, TEAC). The vitamin C standard was also used to express the CEAC value [49].

This ABTS Assay Kit is based on the interaction between antioxidants and the pre-formed green–blue stable radical cationic chromophore, 2,2′-azinobis-(3-ethylbenzothiazoline-6-sulfonate, ABTS•+). In the presence of antioxidants, the oxidized ABTS•+ radical is reduced to ABTS, resulting in a discoloration of the solution, measured by the decrease in absorbance at 734 nm. Antioxidants scavenge ABTS•+ radical cation in a concentration-dependent manner [50].

### 2.9. Data Analysis

Continuous variables were expressed as mean values ± standard deviation (SD). The Kolmogorov–Smirnov test was applied to determine data normality. Differences between physicochemical and biological variables were analyzed using one-way ANOVA and Kruskal–Wallis tests for standard and non-normal distribution variables, respectively. Bivariate correlations (Spearman’s correlation coefficients) were used to assess associations between physicochemical variables and antioxidant parameters. Testing was conducted on several multiple linear regression models, in which differences in resin content were considered dependent variables and the rest of the variables (flavonoid content and antioxidant activity) as predictors. The software package SPSS for Windows version v.26 (IBM SPSS, Inc., Chicago, IL, USA) was used for data analysis. A *p*-value of <0.05 was set as representing statistical significance for all analyses.

## 3. Results

### 3.1. Physicochemical Characterization

As expected, the resins and waxes were the most abundant fractions of all the 31 samples studied. All the results are summarized in Table 1. The mean value for resins was 65.25% for 100 g of propolis, finding higher content in sample 4 with 83.52%; 11 of the 31 propolis samples showed in their composition resin values up to 70%. When waxes were determined, the results ranged between 7.74 ± 0.46% and 39.04 ± 0.67%. The rest of the compounds analyzed were ashes, impurities, and moisture. P3 is highlighted as richer in ash content (2.92 ± 0.21%), almost four times more than the medium value for this fraction (0.85%). When impurities were determined, the range of results was between 0.03% and 0.15%. The water content in the propolis samples, referred to as moisture, was the determination where most differences were found, with a medium value of 16.26 ± 9.39%. No statistically significant differences were observed between samples for the variables analyzed.

### 3.2. Mineral Content

The amounts of the minerals studied in the 31 propolis samples are shown in Table 2. The sodium concentration was below 50 mg/kg for all the samples. Calcium (Ca) and potassium (K) content were the main minerals in propolis samples, while the lowest was for copper (Cu) and sodium, this one (Na) with values under 50 mg/kg (limit detection value). The distribution of the mineral content indicated that sample P3 presented the maximum content for Ca, Fe, and Pb: 2266.01 ± 353.69 mg/kg, 521.95 ± 47.26 mg/kg, and 42,020.36 ± 146.79 µg/kg, respectively. On the other hand, sample P11 presented the minimum content of magnesium (Mg) (87.59 ± 1.24 mg/kg) and manganese (Mn) (2.75 ± 0.13 mg/kg). At the same time, P18 showed the maximum content of mercury (Hg), 39.14 ± 2.05 µg/kg, and zinc (Zn), 442.85 ± 12.38 mg/kg. The content of minerals such as cadmium (Cd) ranged between 14.05 ± 0.11 and 57.04 ± 1.90 µg/kg. The differences between samples were not statistically significant.

### 3.3. Palynological Composition

**Figure 1:**
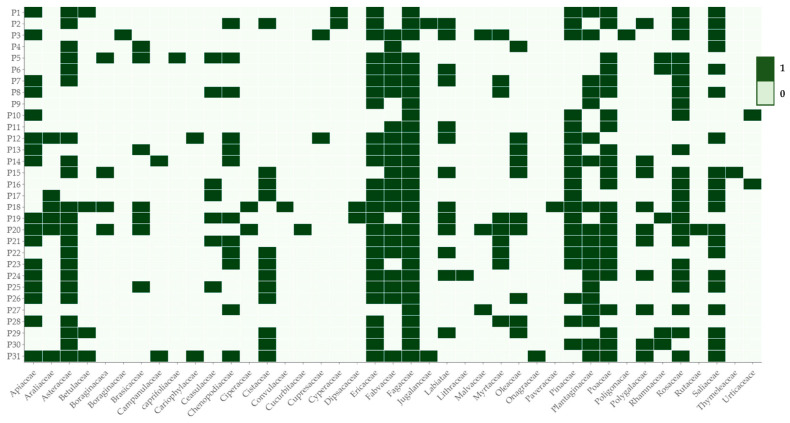
Palynological description of the distribution of pollen grains according to the family taxonomic category for the 31 propolis samples, where 1 means presence and 0 absence.

The palynological study of the propolis samples showed a rich variety of pollen grains. These included *Fagaceae* family, where species such as *Quercus rotundifolia Q. pirenaica* and *Castanea* spp. were identified in 30 of the 31 samples. Families like *Salicaceae*, *Ericaceae*, *Fabaceae*, *Rosaceae*, and *Poaceae* were found in more than 2/3 samples. Conversely, others like *Caprifoliacea*, *Boraginaceae*, or *Thymeleaceae* appeared in only one sample. Figure 1 shows the frequency of occurrence of the identified pollen grains.

### 3.4. Bioactive Compounds

#### 3.4.1. Quantitative Determination of Total Polyphenols and Flavonoid, Flavone, and Flavonol Content

**Figure 2:**
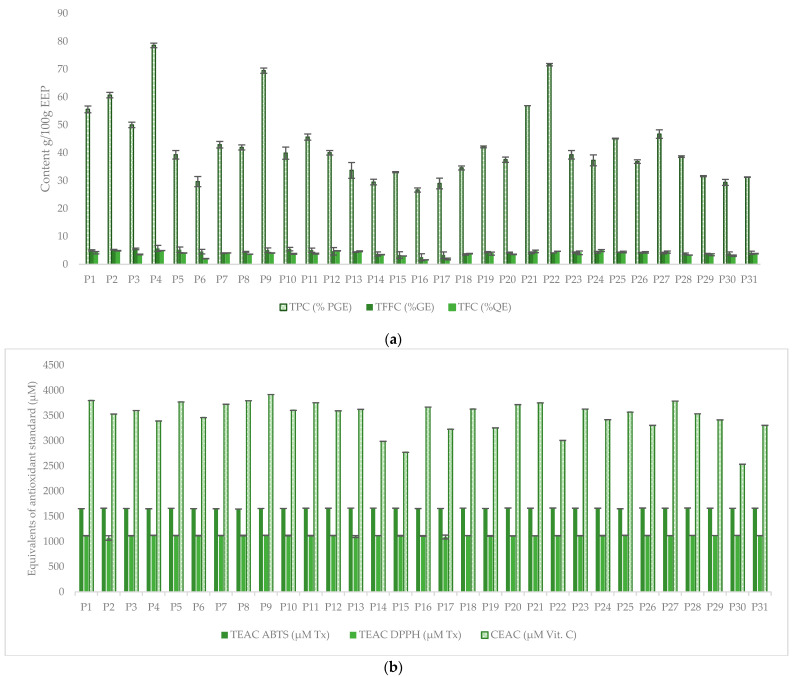
(**a**) Total resin content (RC) and the bioactive compounds expressed as equivalents of pinocembrin and galangin (PGE) for polyphenols (TPC), equivalents of quercetin (QE) for flavonoids (FTC), and equivalents of galangin (GE) for flavonol and flavone content (TFFC); (**b**) results obtained by different scavenging methods to show the antioxidant capacity of each EEP related to standards such as TROLOX and Vitamin C.

The fraction of the main bioactive compounds is shown in Figure 2a. The mean value of total polyphenols (TPC) was 42.72 ± 13.19 expressed as Pinocembrin–Galangin Equivalents in 100 g of raw propolis (RP) sample, although, in P4, a value of 78.54 ± 0.80 g/100 g RP was the result. In addition, a range between 1.64 ± 0.04 and 4.95 ± 0.36 Quercetin Equivalents (QE) g/100 g RP was found for total flavonoids content (TFC). Secondly, the flavone and flavonol content (TFFC) showed remarkably comparable results in all the samples, highlining the P4 and P3 samples with a Galangin Equivalents (GE) average of 5.71 ± 1.07% and 5.53 ± 0.13%, respectively. The comparison between samples revealed no statistically significant differences.

#### 3.4.2. Identification of Chemical Constituents

As expected, the chemical composition of the propolis samples showed a similar chromatographic profile (HPLC). The analyses revealed a diverse range of substances belonging to several groups. More than 50 peaks were identified in all 31 samples and are summarized in Table 3. The chemical characterization involved flavonoids such as apigenin, catechin, chrysin, quercetin, and pinocembrin and phenolic acids, for example, caffeic, ferulic, and coumaric, were characterized. Additionally, caffeic acid phenethyl ester (CAPE) and their derivatives were some phenolic compounds that prevailed regardless of the geobotanical origin.

### 3.5. Antioxidant Activity

The comparison between the results obtained for the ethanolic extraction of propolis showed an extremely high antioxidant capacity compared to the scavenging method used. In both DPPH and ABTS assays, the 31 EEP showed exceedingly high antioxidant power. The results were expressed as Trolox Equivalent Antioxidant Capacity (TEAC) and Vitamin C Equivalents Antioxidant Capacity (CEAC), where units are µM of the standard. Results are detailed in Figure 2b. TEAC, when the DPPH assay was performed, showed mean values of 1114.28 ± 10.39 µM. Otherwise, CEAC ranged between 2535.40 and 3918.18 µM. We did not find statistically significant differences between samples for the antioxidant indicators.

### 3.6. Correlation and Multiple Linear Regression Models

The resin content of propolis samples showed a positive and significant association with both the polyphenols (rho = 0.365; *p* = 0.043) and flavonoid composition (rho = 0.615; *p* = 0.000) and the antioxidant capacity TEAC DPPH (rho = 0.415; *p* = 0.020). In contrast, the association with the wax in the propolis samples was strong although negative (rho = −0.409; *p* = 0.000). The wax content also correlated negatively with the polyphenols and flavonoids. As expected, the ashes correlated positively with impurities and most mineral compounds, whereas the association with the TEACDPPH was negative. The polyphenols exhibited a moderately significant association with the antioxidant profile measured as CEAC (rho = 0.366; *p* = 0.043). Lastly, a multiple regression analysis modeled the relationship between resin composition, flavonoids, and TEAC DPPH values. The significant regression equation using the resin content as a dependent variable explained 61% of the variance (R^2^ = 0.618; F (2, 28) = 22.629; *p* < 0.000; d = 2.299), thus explaining 61% of the variance in CPR global effectiveness (Table 4).

## 4. Discussion

This research offers a complete chemical, palynological, and bioactive profile of propolis obtained from different regions in the northern half of Spain during two harvesting years. Although the chemical characterization of this bee product has been previously reported, the number of studies correlating the physicochemical composition with the bioactive properties of propolis is still scarce [26,27,34]. In this work, we propose a preliminary model that predicts the phenolic content of propolis samples according to their proximal composition and antioxidant capacity [16,51].

First, the physicochemical composition was in line with international recommendations of quality. Per the International Honey Commission (IHC) protocols and specifications [3,41], the acceptable values are 45% minimum for resins, 6% maximum for impurities, 8% maximum for water content, and 5% maximum for ash content. Our results showed that ashes were entirely below the allowable range. Nevertheless, our results displayed a moderate–strong association with several mineral compounds, including Ca, Fe, K, Mg, and Zn, thus suggesting the nutritional value of these propolis [22,52,53]. The broad range of heavy metals found in the raw samples is due to how the beekeepers collected the propolis, the year of harvesting, or even the locations of the apiaries. The harvesting management procedure may explain that two of the 31 samples showed a higher Pb content than mean standard values [54]. It is important to highlight that these results refer to untreated propolis samples. Once processed to their commercial presentation, as ethanolic extracts, the content of heavy metals (Pb, Cd, and Hg) is within the allowable limits of regulatory agencies and the *Codex Alimentarius* standards [23].

The levels of other proximal compounds, like waxes, were within the observed intervals of reported data from different bioclimatic conditions [1,19]. The wax composition was slightly lower compared to other propolis, regardless of the climate region. However, the chemical analyses revealed that resins were the principal component involving more than a third % of the samples presenting values above 70%. This implies similar values concerning propolis collected in the center and east of Europe (Poland, Romania, and Georgia) [55,56,57] and higher values compared to propolis from regions with Mediterranean climatic conditions (Morocco, Portugal, and Greece) [28,58,59].

The chemical characterization can also be associated with the palynological profile of propolis. Thus, the chromatographic analyses allowed the identification of more than 50 phenolic compounds related to the plant origin [4,8,60]. In this sense, the flavonoids and flavonols identified in all samples are generally present in pollen grains from species belonging to the *Fagaceae*, *Salicaceae*, *Ericaceae*, and *Fabaceae* families. For example, the flavonoids (pinocembrin, pinobanksin, chrysin, and galangin) are typical constituents of the exudate sprouts of *Q. rotundifolia*, *Q. pirenaica*, and *Castanea* spp., which are frequent spices growing in the regions where the apiaries were situated. Several phenolic acids and their esters, like caffeic, p-coumaric, ferulic, and CAPE, were abundantly present in *Populus* spp. Typically, from the latitude of Spain, it corresponds to the hive’s location. All of these compounds have been studied as marker compounds [13,25,61]. In addition, the botanical origin, the phenolic content, and the profile of the majority constituents are linked to the bioactive activity, especially to the antioxidant capacity [47,58].

Honeybees mix the pollen grains, parts of the trees, and flowers or secretions of other plants with their fluids to produce propolis. The composition of propolis and the amount of nutrients, especially the antioxidant compounds (phenols, flavonoids, and flavones), are influenced by the botanical family, the richness of flora diversity, the harvesting time, and/or the geographical area [19,25,29,62]. Our findings reflect the correspondence between the botanical source and the biological components. Previous work has established that Mediterranean propolis is abundant in polyphenols, flavonoids, and flavones, while other bioactive substances, such as diterpenoids, isoflavones, and other flavonols, are abundant in non-European samples [20,26,58].

Propolis can be considered a supplement in food because of the antioxidant role conferred mainly by phenolic compounds, such as phenolic acids and flavonoids. The amount of antioxidant compounds depends primarily on the floral source and the geographical origin [9,25].

Despite the geographical origin, climate, botany around the hive, year of collection, and management, among other factors, our results showed that flavonoids and flavonols were in the same range. In contrast, we observed more variations when analyzing polyphenol content [56]. Our samples presented higher TPC than Portuguese or Moroccan propolis (>70%). At the same time, TFC and TFFC were not significantly influenced by the vegetation sources around the hive [28,59,63].

Similarly, the antioxidant activity, measured as Trolox and Vit. C Equivalents of the investigated samples were approximately 100 times higher than other studies [28,38]. These findings confirm that phenolics play a significant role in the antioxidant potential of propolis, which is demonstrated by the inherent link between antioxidant activity and propolis. Therefore, geographical area and the chemical composition derived from numerous botanical sources affect antioxidant properties [13,16,56].

Previous studies attribute the propolis bioactive properties (antibacterial, anti-inflammatory, antioxidant, biomarker, and immunomodulator) to the compounds present in the resinous fraction. In this sense, the resin content is a feasible indicator for evaluating propolis samples’ biological activity and antioxidant capacity. Prior research has found that phenolic content is strongly linked to the antioxidant properties of propolis, even at low resin concentrations [45,47,56]. On the other hand, resins have also been correlated with polyphenols and flavonoids when the wax component diminishes. Both biological activity and antioxidant capacity increase due to the resin fraction of propolis and can affect their natural quality. Considering these factors, it is especially pertinent to look for a pattern correlating resin variety to phenolic components and antioxidant strength. In light of this, our findings indicate that alterations in resin amount are connected to polyphenols and the antioxidant power measured as TEAC DPPH. Data analysis through a multiple regression model produced a statistically significant regression equation using the resin fraction as the dependent variable and polyphenols and TEAC as predictors. The regression analysis showed a consistent pattern, resulting in a statistically significant regression equation (*p* < 0.000) and a Durbin–Watson value of 2.299, indicating the lack of autocorrelation in the sample. Consequently, our results sufficiently support the significance of the analysis to determine the validity and usefulness of the model. These findings may indicate the potential of resins as an indicator of biological activity and the need to identify the influence of phenolic content when characterizing bee products such as propolis.

Although we comprehensively describe the physicochemical characteristics, principal compounds, and bioactive properties of the propolis samples, this study has some limitations. For instance, quantifying specific constituents of the phenolic compounds may help establish more associations between botanical origin, the fractions with biological activity, and antioxidant effects. The knowledge of target antioxidant elements present in the propolis composition might contribute to focusing on the potential of this natural product as a relevant diet supplement with sound applications in the food industry.

## 5. Conclusions

Understanding propolis’ intrinsic quality is essential in the search for natural products suitable in the agroalimentary field. This work shows that the biochemical characterization of propolis provides a valuable trend for predicting their antioxidant capacity. Specifically, the propolis resinous fraction indicates phenolic content and biological activity.

These findings settle propolis as one of the most promising hive products, thus becoming an alternative resource for the sustainability of beekeepers and the apiculture sector. Additionally, transferring an easy-to-use tool for integrating propolis in the routine manufacturing of food products could contribute to developing healthier ingredients and additives.

Therefore, propolis can provide a solid framework for addressing people’s demands while promoting appropriate consumption patterns by developing eco-friendly food supplies.

## Figures and Tables

**Table 1:** Description of physicochemical composition expressed as g/100 g raw propolis.

	Wax (%)	Resin (%)	Ash (%)	Impurities (%)	Moisture (%)
P1	19.80 ± 0.57	60.52 ± 2.66	0.81 ± 0.04	0.06 ± 0.00	12.87 ± 0.01
P2	11.02 ± 4.41	56.04 ± 16.00	0.58 ± 0.03	0.04 ± 0.01	19.20 ± 0.01
P3	18.74 ± 1.91	55.05 ± 1.89	2.92 ± 0.21	0.01 ± 0.01	22.29 ± 0.01
P4	8.68 ± 1.20	83.52 ± 0.85	0.58 ± 0.02	0.06 ± 0.04	1.23 ± 0.01
P5	18.87 ± 0.63	67.46 ± 3.05	0.71 ± 0.02	0.06 ± 0.01	6.96 ± 0.01
P6	31.43 ± 1.15	50.73 ± 6.20	0.92 ± 0.03	0.09 ± 0.00	7.92 ± 0.01
P7	7.64 ± 0.46	71.56 ± 4.41	0.76 ± 0.02	0.07 ± 0.00	12.94 ± 0.01
P8	21.24 ± 0.76	62.20 ± 3.22	0.60 ± 0.02	0.04 ± 0.01	11.97 ± 0.01
P9	9.90 ± 1.22	70.22 ± 1.54	0.75 ± 0.04	0.09 ± 0.00	10.13 ± 0.01
P10	11.91 ± 0.73	71.63 ± 9.17	0.076 ± 0.00	0.04 ± 0.01	11.70 ± 0.01
P11	13.74 ± 0.88	73.79 ± 3.72	0.44 ± 0.00	0.03 ± 0.00	9.04 ± 0.01
P12	21.13 ± 1.61	65.73 ± 1.60	0.92 ± 0.06	0.05 ± 0.01	7.21 ± 0.01
P13	17.23 ± 0.24	66.91 ± 5.25	0.92 ± 0.00	0.05 ± 0.00	9.94 ± 0.01
P14	22.98 ± 1.63	61.08 ± 3.78	0.86 ± 0.01	0.06 ± 0.02	9.08 ± 0.01
P15	27.82 ± 0.70	56.90 ± 1.33	1.47 ± 0.04	0.07 ± 0.00	6.80 ± 0.01
P16	25.19 ± 1.92	48.20 ± 0.99	0.79 ± 0.03	0.15 ± 0.00	10.82 ± 0.01
P17	27.90 ± 4.08	48.50 ± 4.51	0.79 ± 0.02	0.15 ± 0.02	7.81 ± 0.01
P18	15.64 ± 0.88	66.82 ± 1.69	1.77 ± 0.16	0.08 ± 0.00	7.76 ± 0.01
P19	39.04 ± 0.67	47.66 ± 1.46	0.65 ± 0.02	0.05 ± 0.01	7.64 ± 0.01
P20	14.83 ± 1.06	66.86 ± 2.63	1.32 ± 0.57	0.11 ± 0.01	5.99 ± 0.01
P21	11.37 ± 0.81	76.26 ± 1.80	0.67 ± 0.03	0.04 ± 0.00	7.70 ± 0.01
P22	10.64 ± 0.16	74.70 ± 0.86	0.83 ± 0.11	0.04 ± 0.00	9.83 ± 0.01
P23	14.08 ± 0.53	73.88 ± 1.05	0.86 ± 0.02	0.03 ± 0.01	8.18 ± 0.01
P24	11.16 ± 0.61	72.62 ± 1.73	1.01 ± 0.57	0.05 ± 0.01	10.21 ± 0.01
P25	14.05 ± 1.06	72.61 ± 5.08	0.65 ± 0.01	0.05 ± 0.00	7.69 ± 0.01
P26	14.38 ± 1.45	72.51 ± 1.56	0.69 ± 0.03	0.06 ± 0.00	6.42 ± 0.01
P27	13.86 ± 1.10	68.73 ± 1.22	0.45 ± 0.00	0.05 ± 0.00	11.96 ± 0.01
P28	17.66 ± 0.77	67.99 ± 2.44	0.48 ± 0.11	0.03 ± 0.01	13.87 ± 0.01
P29	15.06 ± 0.97	65.19 ± 5.10	0.47 ± 0.03	0.04 ± 0.01	15.27 ± 0.01
P30	16.43 ± 0.42	72.19 ± 5.55	0.33 ± 0.12	0.04 ± 0.00	7.15 ± 0.01
P31	22.17 ± 1.19	67.87 ± 1.70	0.53 ± 0.07	0.04 ± 0.00	5.43 ± 0.01
Mean values	17.58 ± 7.12	65.25 ± 8.50	0.85 ± 0.49	0.06 ± 0.03	16.26 ± 4.87

**Table 2:** Mineral composition of 31 samples of raw propolis collected from the half north of Spain. Ca, Cd, Fe, K, Mg, Mn, and Zn are expressed in mg/kg; Cu, Hg, and Pb are expressed in µg/kg.

	Ca	Cu	Fe	Zn	K	Mg	Mn	Pb	Hg	Cd
P1	739.72 ± 8.51	0.94 ± 0.09	60.63 ± 1.19	27.58 ± 0.78	2198.95 ± 6.93	221.55 ± 3.86	9.93 ± 0.31	4193.15 ± 1099.45	20.72 ± 2.00	30.17 ± 0.07
P2	514.38 ± 9.64	0.52 ± 0.01	50.35 ± 1.87	13.49 ± 0.15	1368.48 ± 9.17	119.50 ± 1.73	14.26 ± 0.13	173.42 ± 17.89	9.86 ± 1.92	24.98 ± 0.16
P3	2966.01 ± 353.69	3.68 ± 0.15	521.95 ± 47.26	319.01 ± 4.90	1840.33 ± 12.81	322.25 ± 14.48	27.62 ± 0.31	42,020.36 ± 146.79	10.35 ± 0.13	39.10 ± 0.11
P4	735.80 ± 45.83	0.80 ± 0.04	61.77 ± 5.54	24.21 ± 0.53	776.89 ± 8.44	104.05 ± 1.47	3.06 ± 0.37	187.44 ± 12.39	7.20 ± 0.23	17.28 ± 0.51
P5	656.94 ± 30.17	0.75 ± 0.01	48.50 ± 1.68	20.99 ± 1.02	1502.15 ± 4.17	256.29 ± 15.73	13.94 ± 0.58	205.18 ± 110.13	6.33 ± 0.56	20.78 ± 0.26
P6	851.16 ± 27.32	2.18 ± 0.05	87.06 ± 2.91	106.41 ± 1.44	2532.08 ± 39.46	321.20 ± 4.38	30.31 ± 0.40	1114.07 ± 106.31	6.45 ± 0.79	22.95 ± 1.28
P7	675.95 ± 12.39	1.05 ± 0.04	43.88 ± 10.60	31.68 ± 2.09	2561.94 ± 79.92	348.38 ± 14.16	13.67 ± 0.81	178.47 ± 4.93	2.98 ± 1.17	18.56 ± 0.48
P8	638.64 ± 53.02	0.93 ± 0.01	78.41 ± 17.53	33.42 ± 0.37	1519.16 ± 25.79	199.88 ± 0.59	13.83 ± 1.64	2258.74 ± 1948.59	2.48 ± 0.30	14.05 ± 0.11
P9	731.23 ± 7.16	1.54 ± 0.11	43.78 ± 2.42	19.57 ± 0.04	2234.26 ± 20.51	362.68 ± 9.20	18.09 ± 0.82	91.52 ± 4.69	4.52 ± 0.69	18.54 ± 1.14
P10	632.82 ± 22.58	0.99 ± 0.08	48.15 ± 2.25	25.49 ± 1.70	2359.82 ± 47.19	299.92 ± 4.87	12.52 ± 0.73	577.40 ± 216.03	2.63 ± 0.07	36.35 ± 0.40
P11	412.43 ± 11.28	3.70 ± 0.14	44.25 ± 1.04	15.81 ± 0.59	1006.92 ± 7.68	87.59 ± 1.24	2.75 ± 0.13	222.22 ± 58.96	1.90 ± 0.08	17.51 ± 1.77
P12	1320.72 ± 118.02	1.64 ± 0.11	213.46 ± 49.78	204.66 ± 36.90	996.58 ± 83.92	155.14 ± 10.47	4.99 ± 0.06	3161.28 ± 1149.33	4.87 ± 0.92	32.25 ± 0.35
P13	1144.17 ± 32.88	0.70 ± 0.01	26.59 ± 2.69	34.39 ± 0.42	3587.01 ± 93.95	268.38 ± 5.29	6.09 ± 0.43	290.24 ± 0.41	1.71 ± 0.32	44.15 ± 1.61
P14	1069.32 ± 115.58	1.18 ± 0.08	167.86 ± 92.12	78.40 ± 6.68	1636.67 ± 78.91	207.37 ± 31.69	7.62 ± 0.60	2653.20 ± 595.31	4.18 ± 0.26	35.32 ± 2.46
P15	919.58 ± 70.29	1.22 ± 0.01	200.27 ± 9.26	27.43 ± 2.17	2247.08 ± 216.69	280.18 ± 19.05	24.01 ± 1.17	450.50 ± 55.20	12.85 ± 1.48	57.04 ± 1.90
P16	683.50 ± 36.96	5.82 ± 3.36	71.44 ± 0.06	65.17 ± 1.85	1592.88 ± 12.32	345.66 ± 8.59	63.27 ± 0.14	746.17 ± 345.72	3.38 ± 0.69	17.10 ± 0.31
P17	750.36 ± 56.90	3.59 ± 0.07	66.57 ± 1.97	68.36 ± 0.47	1755.00 ± 17.45	370.40 ± 27.27	52.29 ± 0.19	455.72 ± 148.48	2.43 ± 0.98	23.23 ± 0.44
P18	2454.39 ± 18.76	3.04 ± 0.36	473.1 ± 13.68	442.85 ± 12.38	1640.94 ± 11.64	304.51 ± 4.93	9.39 ± 0.13	35,252.02 ± 7936.88	39.14 ± 2.05	34.26 ± 2.12
P19	784.14 ± 76.46	0.83 ± 0.05	72.08 ± 5.69	4.20 ± 0.28	1071.86 ± 75.31	195.76 ± 17.76	3.38 ± 0.27	168.12 ± 28.67	5.73 ± 0.81	4.59 ± 0.29
P20	1653.01 ± 10.99	3.37 ± 0.35	302.88 ± 6.80	204.36 ± 34.49	1629.66 ± 92.48	309.14 ± 7.55	13.47 ± 0.41	1531.35 ± 85.34	5.33 ± 0.12	48.24 ± 1.13
P21	676.05 ± 2.85	0.75 ± 0.05	69.01 ± 1.67	115.87 ± 4.07	1656.02 ± 67.55	170.18 ± 2.58	10.46 ± 2.62	4349.00 ± 1067.64	2.21 ± 0.51	19.29 ± 1.19
P22	647.01 ± 52.38	0.73 ± 0.04	61.57 ± 0.03	43.71 ± 6.72	1515.78 ± 21.16	152.98 ± 5.58	8.42 ± 0.99	1799.98 ± 204.40	2.02 ± 0.46	19.32 ± 0.68
P23	646.43 ± 14.55	1.20 ± 0.29	85.02 ± 16.11	79.72 ± 18.87	1589.56 ± 15.02	166.38 ± 1.46	9.47 ± 0.01	3325.59 ± 482.50	2.91 ± 0.01	20.64 ± 0.45
P24	921.90 ± 5.43	0.80 ± 0.23	59.33 ± 5.60	46.47 ± 4.84	2331.06 ± 24.51	230.69 ± 3.26	8.02 ± 0.22	1003.17 ± 103.88	2.35 ± 0.10	19.79 ± 0.15
P25	602.45 ± 8.24	0.97 ± 0.28	74.07 ± 10.97	43.60 ± 5.44	1457.66 ± 1.66	150.97 ± 0.85	10.45 ± 3.19	2729.06 ± 1876.26	2.12 ± 0.64	19.76 ± 0.48
P26	602.04 ± 13.17	0.80 ± 0.01	77.61 ± 2.72	58.59 ± 28.16	1481.58 ± 104.93	146.11 ± 2.16	8.62 ± 0.42	9286.69 ± 9473.28	1.49 ± 1.29	19.47 ± 0.97
P27	598.45 ± 4.23	0.80 ± 0.05	74.07 ± 0.47	49.52 ± 3.59	1483.01 ± 47.77	148.30 ± 2.80	12.07 ± 4.27	2816.14 ± 1547.44	1.96 ± 1.06	20.88 ± 0.30
P28	356.12 ± 11.74	0.47 ± 0.03	40.05 ± 0.00	15.42 ± 1.07	1242.67 ± 20.34	116.27 ± 1.38	6.95 ± 0.23	1004.73 ± 93.24	0.19 ± 1.15	17.57 ± 1.19
P29	345.86 ± 14.15	0.52 ± 0.06	42.17 ± 0.29	17.10 ± 1.05	1214.72 ± 16.40	113.63 ± 6.50	6.85 ± 0.28	1192.38 ± 176.53	1.00 ± 0.40	16.78 ± 1.14
P30	357.14 ± 22.93	0.51 ± 0.06	43.69 ± 1.11	16.50 ± 0.74	1131.38 ± 0.72	111.21 ± 0.07	6.98 ± 0.06	1149.05 ± 43.23	1.64 ± 0.45	17.22 ± 0.53
P31	472.11 ± 22.86	0.82 ± 0.09	32.95 ± 0.81	31.35 ± 0.11	1232.53 ± 33.04	165.02 ± 2.09	8.16 ± 0.02	1180.15 ± 122.82	0.21 ± 1.10	35.63 ± 3.04
Mean	856.56 ± 571.22	1.51 ± 1.29	107.82 ± 120.25	73.72 ± 96.64	1690.15 ± 585.91	217.79 ± 88.18	14.22 ± 13.38	4056.98 ± 9458.83	5.59 ± 7.53	26.58 ± 11.23

**Table 3:** Phenolic identification profile of the raw propolis samples HPLC-DAD-MS/MS.

Peak	RT (min.)	λ (nm)	[M − H] (*m*/*z*)	MS^2^ (*m/z*)	Component
1	6.4	330	179	135	Caffeic Acid
2	9.5	330	163	119	P-Coumaric Acid
3	10.5	330	193	178, 149, 134	Ferulic Acid
4	11.1	330	193	178, 134	Isoferulic Acid
5	15.0	280	433	271, 165	Pinobanksin Glucoside
6	17.4	330	431	268, 239	Genistein Glucoside
7	18.3	360	207	163, 133	3,4-Dimethyl-Caffeic Acid (DMCA)
8	19.4	360	299	284, 255, 227	Methylluteolin
9	21.3	360	329	315, 299, 285	Dimethylquercetin
10	22.4	280	301	179, 151	Quercetin
11	23.7	280	285	267, 253	Methylpinobanksin
12	23.8	360	285	267, 251	Sakuranetin
13	24.4	280	315	301, 271, 255	Methylquercetin
14	27.8	280	299	270, 255	Methylapigenin (Ej. Hispidulin)
15	28.2	280	267	252, 224, 180	Methylchrysin
16	29.1	330	271	177, 151, 119	Pinobanksin Derivative
17	29.5	280	269	225, 180, 149, 117	Apigenin
18	30.8	360	271	253, 197	Pinobanksin
19	32.2	360	285	257, 229, 151	Kaempferol
20	32.8	360	315	301, 151	Methylquercetin
21	34.1	360	299	284, 255, 227	Methylluteolin (Luteolin-Methyl-Ether)
22	35.6	360	329	314, 299, 285	Methoxykaempferol 3-Methyl Ether
23	38.1	360	283	268, 239, 211	Methoxy-Chrysin
24	38.8	330	301	165, 135	Coumaric Acid Derivative
25	41.5	360	315	301, 193, 165, 121	Quercetin-7-Methyl-Ether
26	45.5	360	329	315, 299, 271	Quercetin-Dimethyl-Ether
27	47.0	280	287	193, 181, 166	Pinobanksin-5-Methyl-Ether
28	48.2	330	247	179, 135	Caffeic Acid Prenyl Ester
29	49.7	330	269	168, 161, 134	Caffeic Acid Benzyl Ester
30	50.5	280	253	209, 167	Chrysin
31	51.7	280	257	255, 213, 151	Pinocembrin
32	52.8	360	269	227, 197	Galangin
33a	53.8	330	283	179, 161, 135	Caffeic Acid Phenylethyl Ester (CAPE)
33b	53.8	280	313	271, 253	Pinobanksin-3-O-Acetate
34	55.6	280	283	268, 239	Methoxy-Chrysin
35	58.5	330	295	178, 134	Caffeic Acid Cinnamyl Ester
36	59.0	330	297	179, 161, 135	Caffeic Acid Methyl Phenetyl Ester
37	59.5	360	283	268, 177, 133	Galangin-5-Methyl-Ether
38	59.8	330	551	429, 283, 267, 255	CAPE Derviative
39	60.9	280	327	271, 253	Pinobanksin-5-Methyl-Ether-3-O-Acetate
40	61.0	330	267	163, 145, 119	Coumaric Acid Derivative
41	62.4	330	301	283, 269, 253, 152	Methoxychrysin Derivative
42	63.2	360	421	313, 299	Luteolin 6-C-Pentoside (Arabinoside)
43	63.5	280	271	253, 165, 152	Pinobanksin
44	64.8	330	279	235, 195, 118	P-Coumaric Cinnamyl Ester
45	65.8	280	341	271, 253	Pinobanksin-O-Butyrate
		280	413	251, 179, 161, 135	Caffeic Acid Derivative
46	66.7	280	363	269, 257	Pinocembrin Derivative
47	67.5	280	285	267, 239,192	Pinobanksin-5-Methyl-Ether
48	68.1	360	283	268, 239	Galangin-5-Methyl-Ether
49	68.3	280	521, 271	283, 269	Galangin Methyl Ether Derivative, Naringenin
50	69.8	280	355	271, 255	Pinobanksin-3-O-Pentanoate or 2-Methylbutyrate
53	71.0	330	315	179, 131	Caffeic Acid Derivative
54	73.6	280	293	197, 185	P-Methoxy Cinnamic Acid Cinnamyl Ester

RT: retention time, λ: wavelength; MS: mass spectrometer.

**Table 4:** Multiple linear regression analysis to model the relationship between differences in resins and antioxidant parameters (polyphenols and TEACDPPH values).

Dependent Variable: Resins	Unstandardized	Coefficients	Standardized Coefficients Beta		
	B	Standard Error		Statistic t	Significance
Constant	250.849	110.643		−2.267	0.031
Flavonoids	7.468	1.218	0.717	6.131	0.000
TEAC DPPH	0.258	0.099	0.304	2.602	0.015

TEAC: Trolox Equivalents Antioxidant Capacity. DPPH: 2,2-diphenyl-1-picrylhydrazyl method.

## Data Availability

The data used to support the findings of this study can be made available by the corresponding author upon request.
